# Rapunzel Syndrome Resulting in Multiple Sites of Simultaneous Intussusception

**DOI:** 10.7759/cureus.90733

**Published:** 2025-08-22

**Authors:** Eric C Morey, Caitlin Brandle, Stephen Schaffner, Dallas Q Sturdevant

**Affiliations:** 1 Radiology, Augusta University Medical College of Georgia, Augusta, USA

**Keywords:** bezoar, intussusception, rapunzel syndrome, trichobezoar, trichotillomania

## Abstract

We present a case of Rapunzel syndrome in a nine-year-old girl with nonverbal autism, pica, and trichotillomania, who presented for acute worsening of abdominal pain and vomiting. Imaging revealed a bezoar extending from the stomach through most of the small intestine, resulting in multiple small bowel-small bowel intussusceptions. Open laparotomy confirmed the presence and enabled the removal of gastroenteric trichobezoars. Trichobezoars are seen most often in adolescent females with a history of trichotillomania and trichophagia. Trichobezoars can cause serious complications, including obstruction, perforation, and intussusception, most notable in the setting of Rapunzel syndrome. Abdominal computed tomography is the preferred imaging modality for diagnosis and surgical planning. The current case highlights the unique radiologic features of extensive bezoar disease and emphasizes the importance of early recognition and definitive operative management to reduce morbidity.

## Introduction

Bezoars are aggregates of indigestible food products that form mass-like conglomerates within the gastrointestinal tract. Trichobezoars, formed from hair products, are a rare yet significant cause of small bowel obstruction and intussusception in children, seen most often in females with a history of trichotillomania and trichophagia. Symptoms of gastrointestinal bezoars vary widely, ranging from asymptomatic to the acute abdomen [1]. Prior to the onset of more serious complications, trichobezoars frequently present with nonspecific symptoms such as abdominal pain and constipation and are therefore commonly overlooked as a possible etiology in the workup of abdominal pain. We present a case of a nine-year-old female with a history of nonverbal autism and trichotillomania found to have a large trichobezoar resulting in multiple small bowel intussusceptions. 

## Case presentation

A nine-year-old girl with a history of nonverbal autism, pica, trichotillomania, and alopecia presented to the emergency department for sudden onset abdominal pain and multiple episodes of non-bloody emesis. The patient had been experiencing several months of constipation as well as recurrent, episodic abdominal pain. She was hospitalized for severe symptoms four months prior. At that time, abdominal radiographs revealed a large stool burden with dense material throughout the right hemiabdomen and without evidence of bowel obstruction. Medical management was pursued with nasogastric tube placement and bowel regimen, resulting in symptomatic improvement. At discharge, intermittent abdominal pain and constipation were managed on an outpatient basis with daily laxative therapy.

The patient’s symptoms were relatively well controlled until the morning of presentation to the emergency department. The patient indicated she had severe abdominal pain that persisted after a large bowel movement following a rectal suppository. Vitals were unremarkable apart from mild tachycardia of 114 beats per minute. Physical examination was significant for “firmness” in the epigastrium and left upper quadrant without overt distention. The patient endorsed diffuse abdominal tenderness with guarding. Laboratory analysis was within normal limits. A constellation of findings raised a broad clinical differential, including small bowel obstruction, bezoar, volvulus, and constipation.

**Figure 1:**
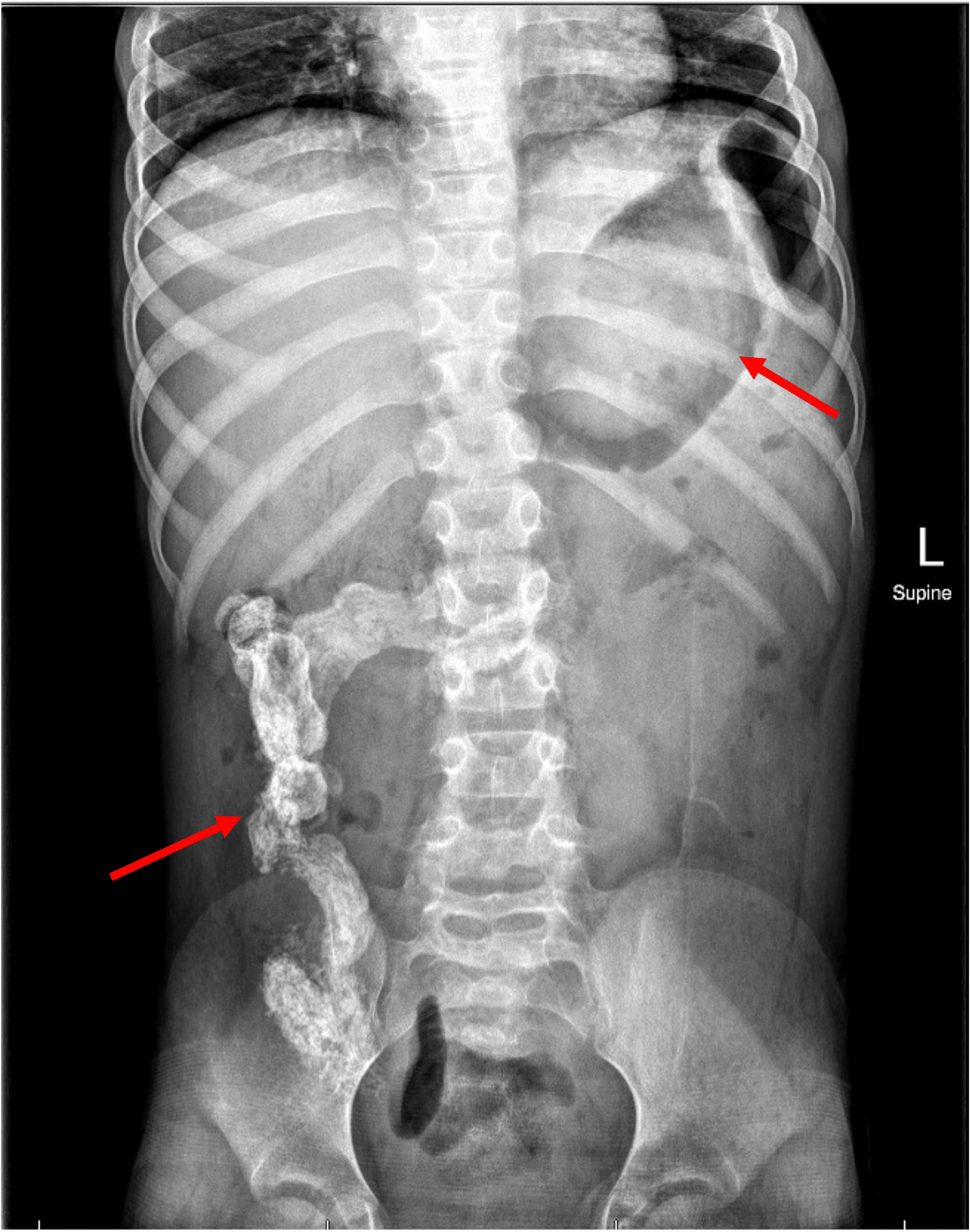
The supine anterior-posterior abdominal radiograph demonstrates a rounded radiodensity within the stomach, with near-circumferential gastric gas concerning for a bezoar. There is a large amount of stool throughout the colon, with radiopaque contents in the cecum, ascending colon, and transverse colon at the hepatic flexure. The bowel gas pattern is nonspecific, with a paucity of bowel gas and no evidence of free intraperitoneal air

An abdominal radiograph was obtained and demonstrated a rounded radio-opacity in the stomach, large colonic stool burden with similar appearance of tubular radiopaque contents projecting over the right hemicolon. There was a relative paucity of bowel gas, which was nonspecific and could not exclude obstruction (Figure 1).

**Figure 2:**
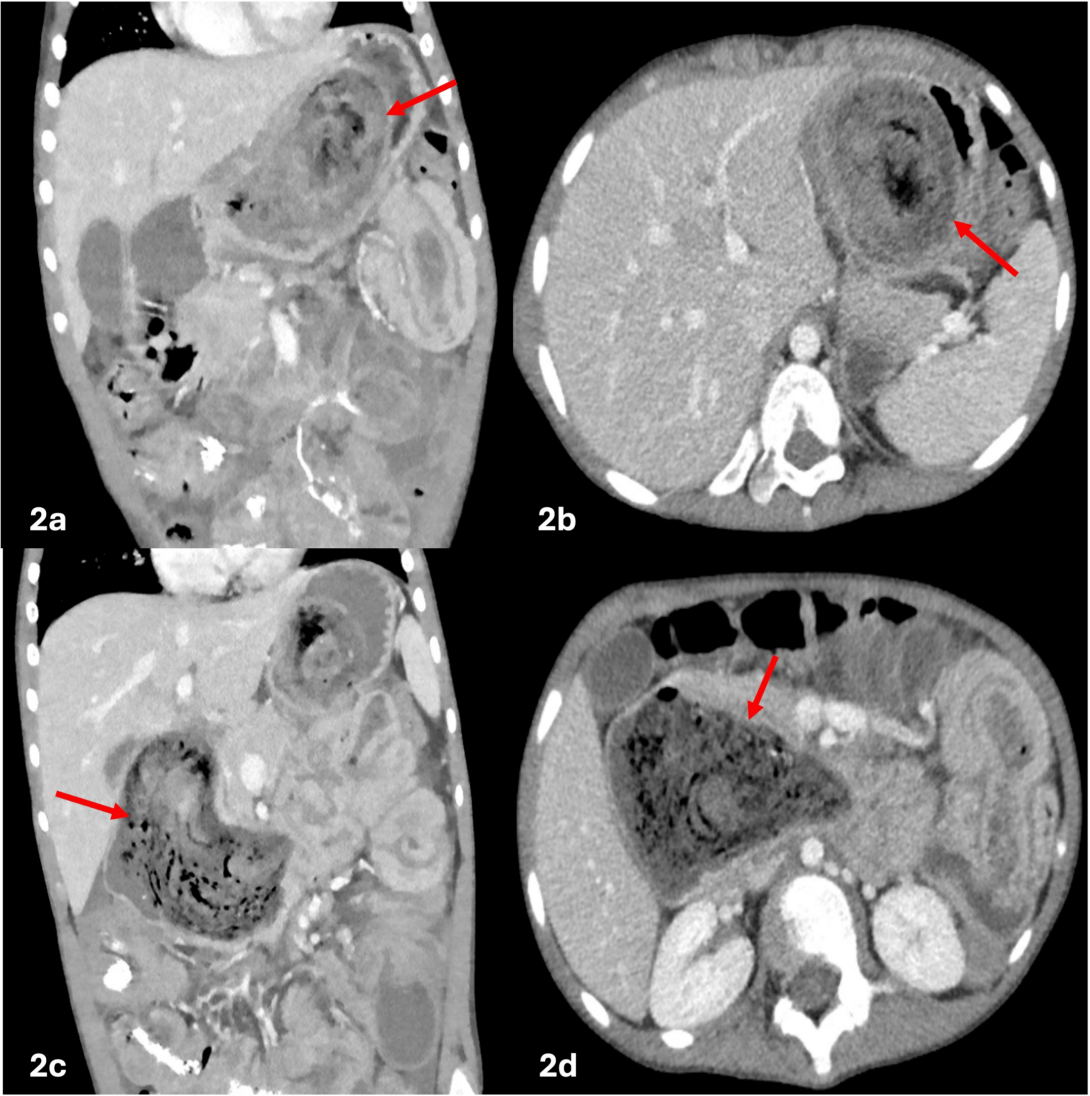
Contrast-enhanced CT images of the abdomen in coronal (2a and 2c) and axial (2b and 2d) views show a large heterogeneous intraluminal mass within the stomach and duodenum, with lamellated, non-enhancing components and intermixed gas, concerning for a trichobezoar CT: computed tomography

**Figure 3:**
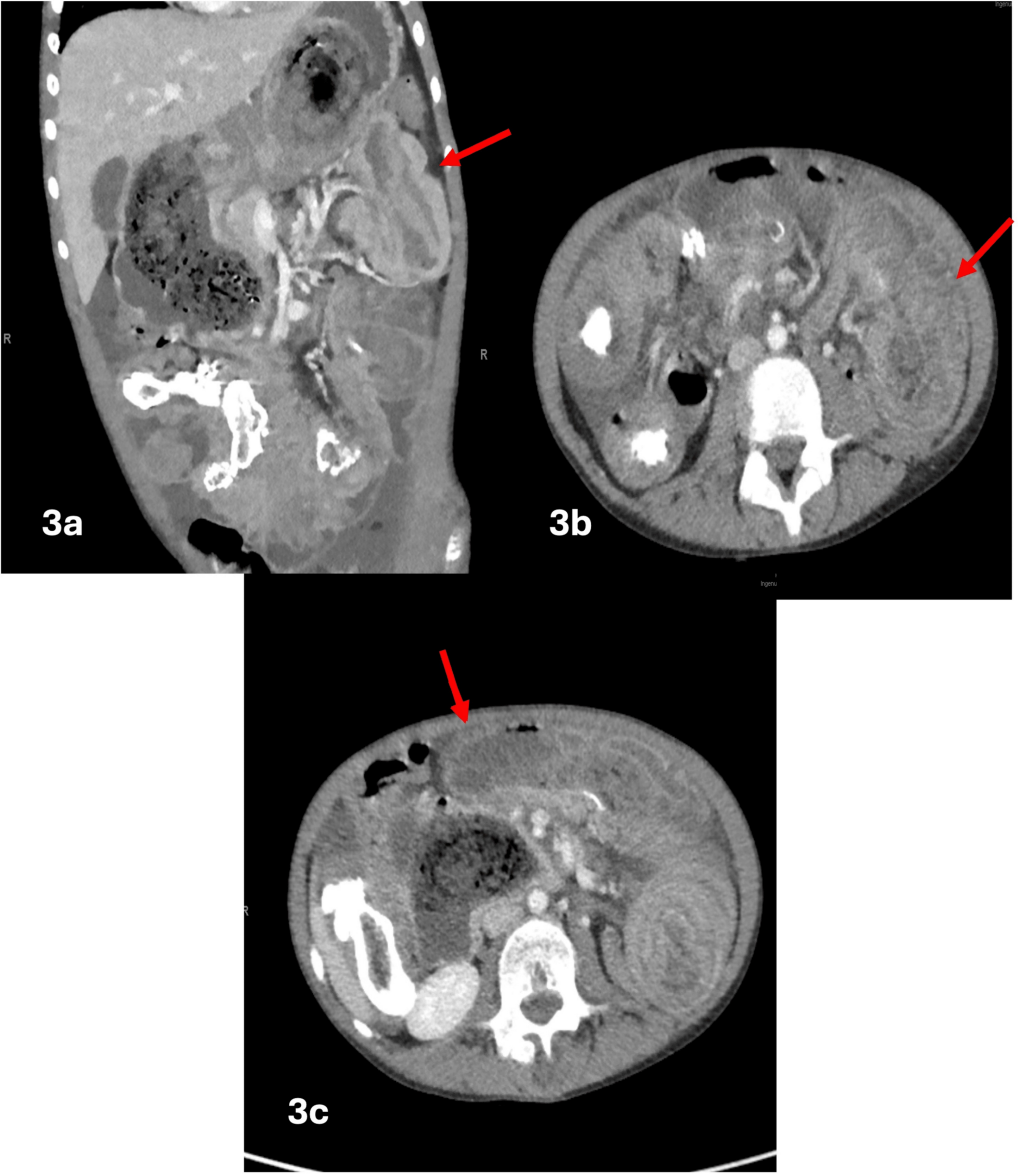
Contrast-enhanced CT images of the abdomen in the axial (3b and 3c) and coronal (3a) planes demonstrate multifocal regions of small bowel telescoping, with a resultant bowel-within-bowel configuration characterized by concentric enhancing bowel walls separated by mesenteric fat. This yields a targetoid appearance in the short-axis plane and a sandwich or pseudokidney sign in the longitudinal axis. Associated bowel wall edema extends from the lower left abdominal quadrant, crosses the mid-abdomen, and terminates in the lower right abdominal quadrant CT: computed tomography

**Figure 4:**
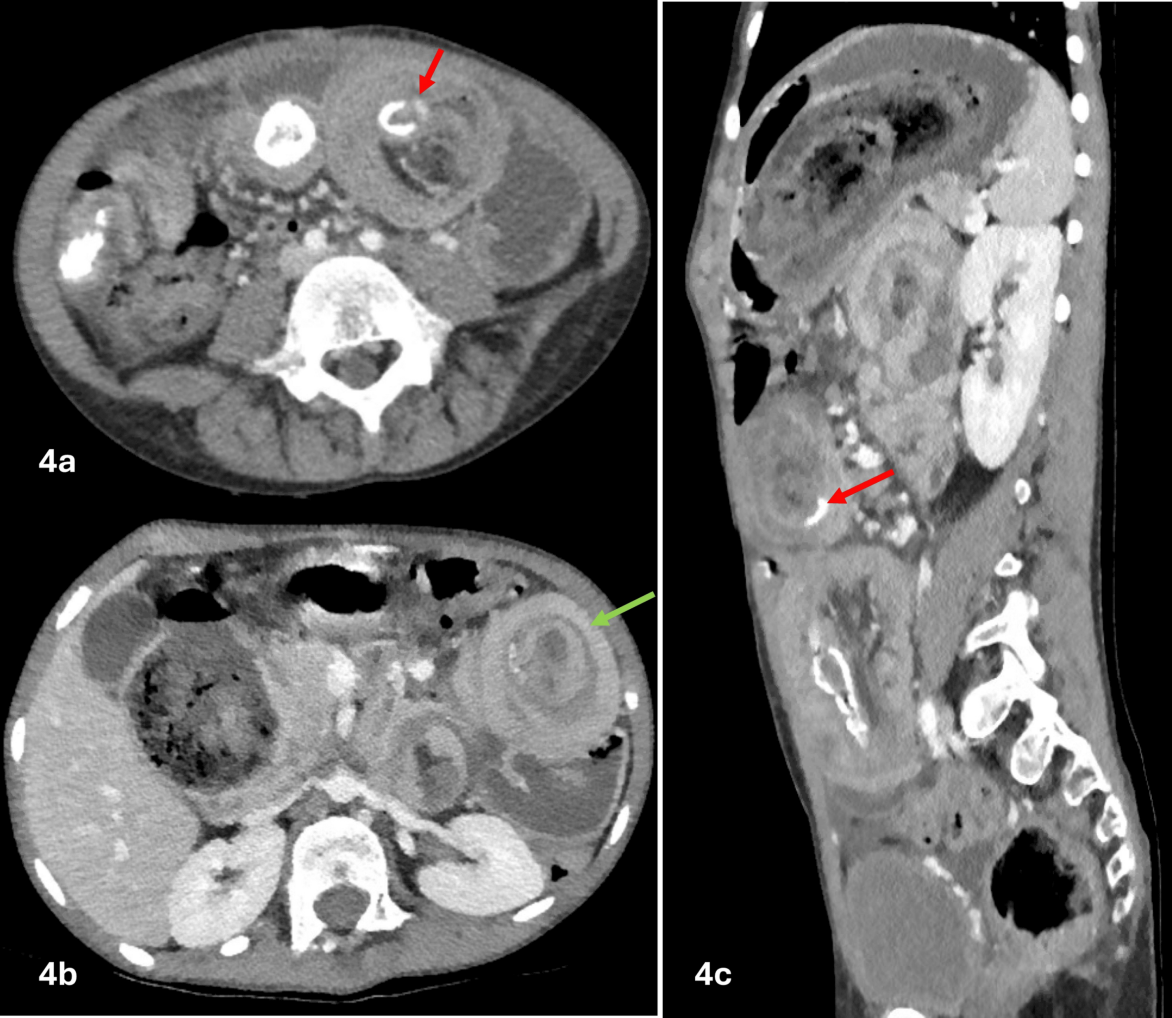
Contrast-enhanced CT images of the abdomen in axial (4a and 4b) and sagittal (4c) views demonstrate a second small bowel-small bowel intussusception, producing the characteristic CT target sign of concentric layers of enhancing bowel wall separated by mesenteric fat (green arrow). Several foci of concreted stool and undigested material are noted, acting as lead points (red arrows) CT:  computed tomography

Contrast-enhanced computed tomography (CT) showed a large trichobezoar extending from the stomach to the jejunum. The jejunal bezoar tail acts as a lead point for a long-segment small bowel-small bowel intussusception, resulting in telescoping of the small bowel and producing the characteristic "bowel-within-bowel" configuration. A second discontinuous small bowel-small bowel intussusception was identified within the midline lower abdomen extending to the right lower quadrant, suspected to be secondary to concreted stool and bezoar acting as a lead point (Figures 2, 3, 4). 

**Figure 5:**
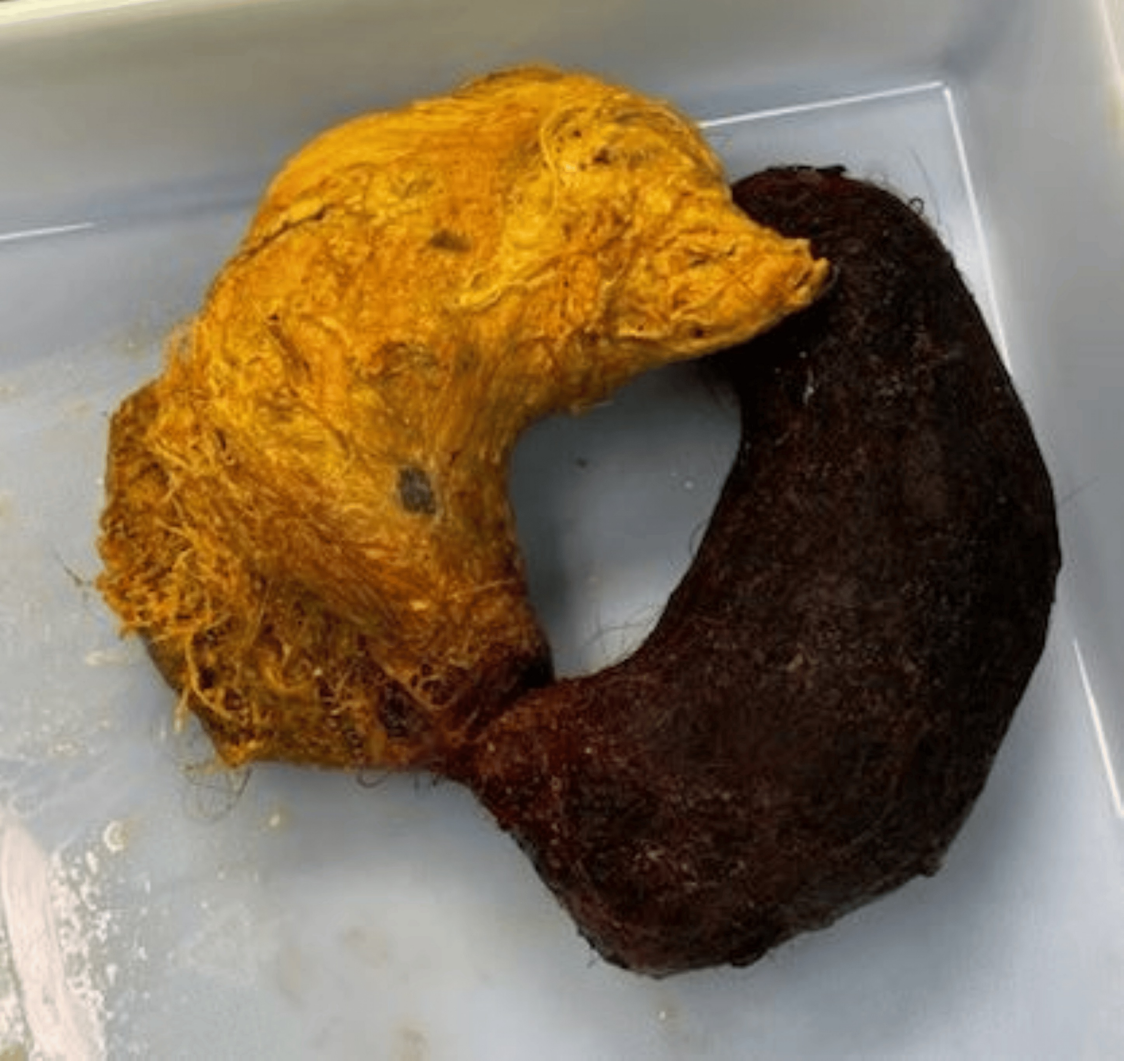
Intraoperative specimen of a gastric trichobezoar

**Figure 6:**
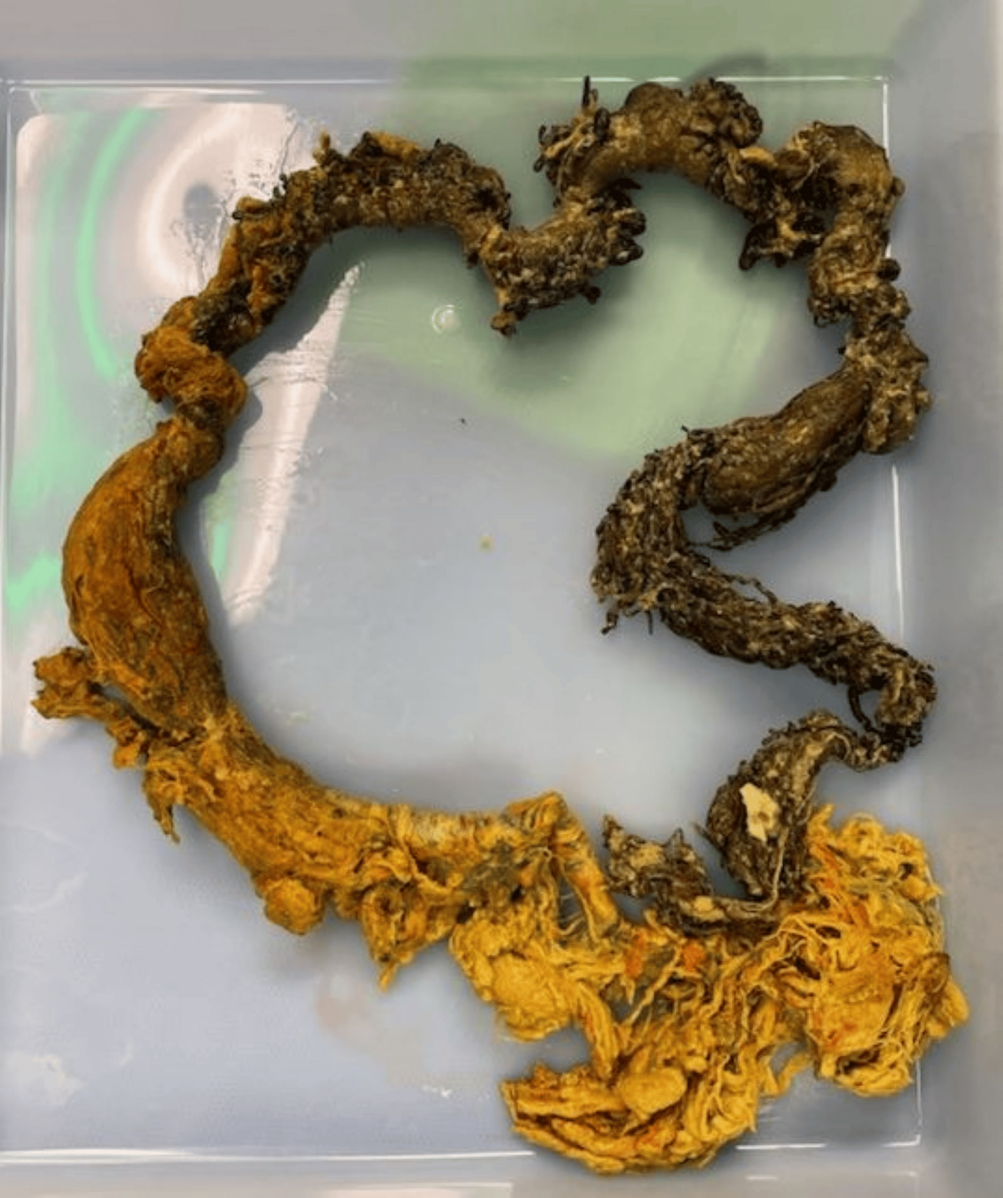
Intraoperative specimen of a small intestine bezoar

An exploratory laparotomy confirmed foreign material present within the stomach and small bowel. A large, continuous trichobezoar within the small bowel coursed from the ligament of Treitz to the proximal ileum, causing multiple discontinuous small bowel-small bowel intussusceptions. The intussusceptions were reduced, and the bezoar was transected into two portions, which were subsequently milked through a mid-jejunal enterotomy and a proximal ileal enterotomy. The bezoar was then inspected and found to consist of fabric, strings, metallic pieces, and hair (Figure 5 and Figure 6). Attention was then turned towards the stomach, where an anterior, longitudinal incision was made, revealing a large gastric trichobezoar extending to the duodenum without evidence of viscus perforation. The duodenal bezoar was milked proximally through the pylorus and removed intact with the gastric bezoar. 

The postoperative course was uncomplicated, and the patient received a seven-day course of intravenous ceftriaxone, fluconazole, and metronidazole. Upon return of bowel function and oral intake tolerance, the patient was discharged. At one-month follow-up, the patient and family members expressed significant improvement in abdominal symptoms and favorable recovery following surgical treatment.

## Discussion

Bezoars are classified into four types based on their constituent components: phytobezoar (indigestible plant products), trichobezoar (hair), lactobezoar (milk protein), and pharmacobezoar (concretion of medications) [1]. Trichobezoars predominantly occur in females of early adolescence with a history of trichotillomania [2,3]. Ingested hair material is usually confined to the stomach but may possess a tail that may extend proximally into the esophagus or distally into the small intestine [4]. Rapunzel syndrome is a rare manifestation of trichobezoar, first named by Vaughan et al., presenting with abdominal pain and signs of obstruction secondary to a gastric trichobezoar and bezoar tail that extends past the pylorus [5]. The clinical diagnosis of gastrointestinal bezoar is difficult due to nonspecific symptoms such as constipation, bloating, nausea, vomiting, anorexia, dysphagia, halitosis, and weight loss. Findings that may heighten provider suspicion for gastrointestinal bezoar, specifically trichobezoar, include patchy regions of alopecia, history of psychiatric illness, and occasionally a palpable mass within the epigastrium, as in the current case [1]. 

Imaging is pivotal in the diagnosis of bezoar-associated intestinal obstruction. The classical abdominal radiograph findings of a bezoar include gastric distension accompanied by an intragastric mottled gas pattern secondary to air within and around the organized ingested material. Radiographs alone are often nonspecific and not able to definitively identify bezoars, necessitating further evaluation with abdominal CT [6,7]. On CT, trichobezoars appear as heterogeneous masses filling the stomach lumen with surrounding gastric fluid contents, denoting the absence of wall attachment [8]. Additionally, CT affords more feasible identification of trichobezoar extension into the small bowel [8]. The current case highlights the added utility of CT in identifying complications of bezoars (e.g., intussusception), which are often radiographically occult but carry a significant risk of mortality.

Delay in diagnosis and management of trichobezoar results in increased bezoar size as well as increased risk for complications, most commonly in the form of gastric or bowel perforation [9]. Additional complications of trichobezoar reported in the literature include intussusception, obstructive jaundice, protein-losing enteropathy, pancreatitis, and, rarely, death [2,9-15]. The current case highlights the extent of intussusception that may result from a trichobezoar. Rapunzel syndrome is identified in the majority of previously published studies regarding trichobezoar-induced intussusception. Trichobezoars limited to the small intestine without a gastric component are less commonly observed; however, they may also result in intussusception [16]. Multifocal regions of intussusception in the setting of Rapunzel syndrome, as demonstrated by the current case, occasionally occur, potentially increasing disease morbidity and the risk of mortality [17-19].  

While other types of bezoars without evidence of bowel obstruction may be managed conservatively, surgical management of trichobezoars is recommended, given that they are not amenable to chemical dissolution [20]. Conventional laparotomy is the preferred method for definitive treatment of trichobezoars, while laparoscopic and endoscopic management is practiced less frequently and with lower success rates [3,9]. Particularly in the setting of Rapunzel syndrome, when the bezoar extends beyond the pylorus, attempts at removal using other strategies carry increased risks due to the potential for dislodgement of bezoar fragments during endoscopy and prolonged operative times with laparoscopy [9]. Furthermore, the presence of complications such as intussusception or perforation necessitates careful inspection of the entire length of bowel, which is far more feasible with an open surgical approach. Following surgical removal, trichobezoars and Rapunzel syndrome may recur if underlying risk factors are not addressed through psychiatric management, typically involving habit reversal therapy and, in some cases, pharmacologic treatment [17,20].

## Conclusions

Trichobezoar is an uncommon cause of pediatric abdominal pain and should be considered in the differential diagnosis for patients with a history of trichotillomania, especially if there is concomitant trichophagia and/or pica. The radiologist plays a crucial role in diagnosis and patient outcomes by encouraging the collection of a detailed ingestion history from the patient or family members. Radiographic findings of gastrointestinal bezoars may demonstrate a gastric intraluminal mass; however, they are often nonspecific, requiring advanced imaging such as CT to fully delineate the extent of the bezoar and any potential complications. Trichobezoars are associated with serious complications and are optimally managed with surgical removal of the foreign material.

## References

[1] Sanders MK (2004). Bezoars: from mystical charms to medical and nutritional management. Practical Gastroenterology.

[2] Baheti AD, Otjen JP, Phillips GS (2017). A hairy situation: trichobezoar presenting with intussusception, and intestinal and biliary perforation in a child. Radiol Case Rep.

[3] Fallon SC, Slater BJ, Larimer EL, Brandt ML, Lopez ME (2013). The surgical management of Rapunzel syndrome: a case series and literature review. J Pediatr Surg.

[4] Mezoff EA, Mezoff AG (2021). 29 - Bezoars. Pediatric Gastrointestinal and Liver Disease.

[5] Vaughan ED Jr, Sawyers JL, Scott HW Jr (1968). The Rapunzel syndrome. An unusual complication of intestinal bezoar. Surgery.

[6] Ripollés T, García-Aguayo J, Martínez MJ, Gil P (2001). Gastrointestinal bezoars: sonographic and CT characteristics. AJR Am J Roentgenol.

[7] Verstandig AG, Klin B, Bloom RA, Hadas I, Libson E (1989). Small bowel phytobezoars: detection with radiography. Radiology.

[8] Gayer G, Jonas T, Apter S (1999). Bezoars in the stomach and small bowel-CT appearance. Clinical Radiology.

[9] Gorter RR, Kneepkens CM, Mattens EC, Aronson DC, Heij HA (2010). Management of trichobezoar: case report and literature review. Pediatr Surg Int.

[10] Ventura DE, Herbella FA, Schettini ST, Delmonte C (2005). Rapunzel syndrome with a fatal outcome in a neglected child. J Pediatr Surg.

[11] Mehta MH, Patel R (1992). Intussusception and intestinal perforations caused by multiple trichobezoars. J Pediatr Surg.

[12] Schreiber H, Filston HC (1976). Obstructive jaundice due to gastric trichobezoar. J Pediatr Surg.

[13] Hossenbocus A, Colin-Jones DG (1973). Trichobezoar, gastric polyposis, protein-losing gastroenteropathy and steatorrhoea. Gut.

[14] Shawis RN, Doig CM (1984). Gastric trichobezoar associated with transient pancreatitis. Arch Dis Child.

[15] Antunes H, Barroso C, Faria C, Correia-Pinto J (2020). Rapunzel syndrome: the pathway for a prompt diagnosis. Arch Dis Child.

[16] Won MM, Sacks MA, Leigh R, Mendez YS, Goodman LF, Tagge E, Radulescu A (2022). An unusual case of primary ileal trichobezoar causing intussusception. Am J Case Rep.

[17] Prasanna BK, Sasikumar K, Gurunandan U, Sreenath GS, Kate V (2013). Rapunzel syndrome: a rare presentation with multiple small intestinal intussusceptions. World J Gastrointest Surg.

[18] Min KJ, Tchah H, Kim SM, Choi JY (2019). A rare presentation of Rapunzel syndrome with multiple small bowel intussusceptions. Pediatr Emerg Med J.

[19] Raghu V, Nagadi AN, Kumar AC (2018). Rapunzel syndrome and small bowel intussusceptions due to a cotton thread Bezoar: a case report. J Gastron Adom Radiol.

[20] Jensen AR, Trankiem CT, Lebovitch S, Grewal H (2005). Gastric outlet obstruction secondary to a large trichobezoar. J Pediatr Surg.

